# Effects of Decompressive Surgery on Prognosis and Cognitive Deficits in Herpes Simplex Encephalitis

**DOI:** 10.1155/2007/534982

**Published:** 2008-03-05

**Authors:** Ipek Midi, Nese Tuncer, Ahmet Midi, Aynur Mollahasanoglu, Deniz Konya, Aydın Sav

**Affiliations:** ^1^Department of NeurologyMarmara University HospitalTurkey; ^2^Department of PathologyMarmara University HospitalTurkey; ^3^Department of NeurosurgeryMarmara University HospitalTurkey

**Keywords:** Herpes simplex encephalitis, decompressive craniotomy, anterior temporal lobe, cognition

## Abstract

Herpes simplex encephalitis (HSE) is a serious viral infection with a high rate of mortality. The most commonly seen complications are behavioral changes, seizures and memory deficits. We report the case of a 37-year-old man with HSE in the right temporal lobe and a severe midline shift who was treated with acyclovir. The patient underwent anterior temporal lobe resection. Although HSE can cause permanent cognitive deficits, in this case, early surgical intervention minimized any deficit, as determined by detailed neuropsychological examination. Surgical decompression is indicated as early as possible in severe cases. This case report emphasizes the effect of surgical decompression for HSE on cognitive function, which has rarely been mentioned before.

## 1. Introduction

Herpes simplex encephalitis (HSE) is the most common form of acute viral encephalitis [6].Antiviral therapy (acyclovir) should be started whenever HSV is suspected, because of the high incidence of mortality [6, 20]. Cognitive deficits, especially memory loss and frontal dysfunction with behavioral problems, are commonly seen in affected patients, even with early antiviral therapy. Moreover, brain edema resulting in tentorial herniation can develop and threaten the patient's life, despite adequate antiviral treatment [6].

## 2. Case report

A 37-year-old man, presented to the hospital, suffering from severe headache of one-week duration that did not respond to analgesics. At the time of admission, he was conscious, but in a sleepy state, mildly cooperative with limited attention, had difficulty in communicating, and was speaking only a few words, in a mildly dysarthric way. (Glasgow Coma Scale: 11/15, GCS: verbal response = 3, eye response = 3, motor response = 5). His Mini- Mental State Examination [7] score was 12/30. In the orientation part, he did not know the day, date or place. He could not remember words after a short period of time. He could not do the calculations, nor write a sensible sentence. He could not copy the figure.

His body temperature was 37.3°C. Neck stiffness was present. His pupils were equal in size and reactive to light. He had moderate right-sided weakness and

ISSN 0953-4180/07/$17.00 © 2007 – IOS Press and the authors. All rights reserved plantar reflexes were bilaterally unresponsive. He was unable to get up and walk. Cranial MRI performed on the first day of admission revealed a hyperintense lesion in the right temporal lobe, with brain edema on T2-weighted images. Acyclovir treatment was started at a dose of 30 mg/kg/24 h, because HSE was suspected and dexamethasone treatment was begun with 6 $\times$ 8 mg IV, following the MRI findings. Blood hematological values were as follows: leucocyte count was 18900/L (normal = 4000–10000), hemoglobin 15.9 g/dL (normal = 12–17) and hematocrit 47.1% (normal = 36–50%). Liver, kidney, and electrolyte values were within normal limits. Lumbar puncture was performed and CSF material was obtained. Opening pressure was 230 mm H2O. The CSF protein content was 250 mg/dL. The CSF glucose level was normal. CSF examination also showed increased cell counts, with lymphopleocytosis. A PCR test for HSV was positive. An EEG showed slowing activity on the right side.

On the third day of admission, his neurological condition deteriorated progressively and he finally lost consciousness. GCS scores were 5/15 (GCS: verbal = 1, eye = 1, motor = 3) Pupils were anisocoric. Massive brain edema, increased midline shift, trans-tentorial herniation, and compression of the brain stem were seen on CT scan (Fig. 1). After a neurosurgical consultation, surgical management for brain stem decompression was decided. A decompressive craniotomy and anterior temporal lobe resection were performed; solid tissue was sent for pathological examination. Histopathologically, intracytoplasmic eosinophilic inclusions distorting the nucleus in neurons were seen, consistent with the diagnosis of HSV infection.

On the first day after the operation, GCS scores were 8/15 (GCS: verbal = 2, eye = 2, motor = 4). The patient's clinical and radiological status improved and GCS increased to 10/15 (GCS: verbal = 3, eye = 2, motor = 5) on the sixth post-operative day, and he was followed for one month as an in-patient. During this time, he could speak, eat by himself, and began to walk again with support. GCS was 15/15 (verbal = 5, eye = 4, motor = 6). He had still mild hemiparesis on the left side, but his cranial nerves were intact.

After the patient was discharge from the hospital, limited neuropsychological tests were performed one month later because he was not cooperative. He presented with apathy and psychomotor slowness, but appropriate social behavior. The cognitive profile showed moderate memory deficits, especially in anterograde tests, mild deficits in visuospatial function, and mild deficits in planning and abstraction in tests for executive function. Praxis and speech function were preserved.

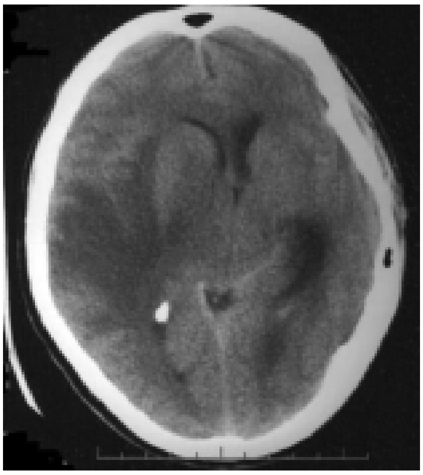

Fig. 1. Non-enhanced cranial CT shows a large low-density region in the right temporal lobe and severe midline shift. Compression of the right lateral ventricle and the brain stem can also be seen.

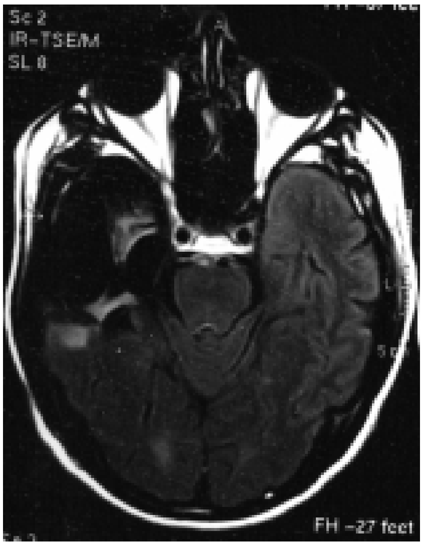

Fig. 2. Axial T1 weighted MRI scan reveals encephalomalacic region in the temporal lobe and total resolution of the compression.

At one-year follow-up, the patient's neurological exam was normal and an encephalomalacic region was seen in his cranial MRI (Fig. 2). He was working at his own company and a detailed neuropsychological exam was performed (Table 1). All of these tests had been studied with respect to their psychometric properties in the Turkish culture [10]. Serial tests in Turkish version for evaluating executive functions were performed (the cut off values of some of these tests in Turkish population is reported in Table 1). He could repeat seven digits in a forward sequence and five digits backwards in the Digit Span Test [18]; this performance

Table 1
Neuropsychological test results of patient one year after operation

| Wechsler memory scale- V digit span subtest [18] | | Normal values | WAIS-R III | | Normal values |
|---|---|---|---|---|---|
| Digit forward (DF) | 7 | (7 ± 2) | Similarities and proverb interpretation tests [18] | **mild impairment** | |
| Digit backward (DB) | 5 | (DF)-(DB) ⩽ 2 | | | |
| | | | California verbal learning test [5,10,12] | | |
| Wechsler memory scale- III digit backward [19] | | | List A trials 1–5 total | **46** | (57.33 ± 7.28) |
| Counting backward from 20 to 1 | 5 seconds | < 35 seconds | List A trial 1 | **4** | (7.87 ± 1.81) |
| Days backward | 3 seconds | < 12 seconds | List A trial 5 | 13 | (13.7 ± 1.58) |
| Months backward | 15 seconds | < 45 seconds | List B | 5 | ( 7.03 ± 2.06) |
| Counting by 3’s | 15 seconds | < 50 seconds | List A short-delay free recall | 11 | (12.8 ± 2.01) |
| | | | List A short-delay cued recall | 14 | (13.8 ± 1.67) |
| | | | List A long-delay free recall | 13 | (13.63 ± 1.79) |
| Luria alternating sequences task [11] | normal | | List A long-delay cued recall | 14 | (14.1 ± 1.71) |
| Construction (clock drawing test) [19] | normal | | Perseverations (free and cued recall total) | 1 | (4.67 ± 4.05) |
| Stroop test [10,15,17] | | | Intrusions (free and cued recall total) | 0 | (3.6 ± 4.36) |
| Spontaneous corrections | 2 | (0.95 ± 1.23) | Recognition | 14 | (15.57 ± 0.9) |
| Time differences | 41 | (40 ± 11) | Discriminability | 98 | (98.4 ± 3.54) |
| Errors | 0 | (0.25 ± 0.72) | False positives | 1 | (0.33 ± 0.84) |
| Wisconsin card sorting test [8,10] | | | Response bias | **−0.39** | (−0.01 ± 0.11) |
| right responses | 81 | (71.40 ± 10.03) | | | |
| wrong responses | 35 | (34.61 ± 21.37) | Wechsler memory scale- VI | | |
| | | | Visual reproduction subtest [10,12,19] | | |
| category number | 6 | (4.98 ± 1.59) | Immediate recall | **8** | (11.73 ± 2.53) |
| perseverative error | 19 | (19.39 ± 13.57) | Delayed recall | 10 | (11.27 ± 2.95) |
| % of perseverative error | 16.4% | (15.48 ± 7.65%) | multiple choice form | | (12.25 ± 1.89) |
| verbal fluency (KAS- in Turkish version) [10] | **36/ 3 min** | (51 ± 11) | Rey complex figure copy [13,14] | 32 | (32.2 ± 4.237) |
| category fluency (animal) [10] | **21/min** | (27 ± 5) | Delayed recall | **10** | (18.7 ± 5.609) |

was normal, indicating adequate functioning of attention, short term memory, and working memory. In the Luria Alternating Sequences Task [11], he was normal. The results of both the Stroop Interference Test [10,15, 17] and the Clock Drawing Test [19] were within normal ranges. In the Wisconsin Card Sorting Test [8,10] he achieved six categories and 81/116 correct responses, and 19 (16.4%) perseverative errors (normal performance). Both verbal and categorical fluency were mildly impaired. On the WAIS III 'Similarities and Proverb Interpretation Tests' [18], reasoning and abstraction were mildly impaired. Both verbal and visual memory were tested [5,10,12,18]. While recall was slightly impaired, recognition performance was preserved. Complex perceptual and spatial processing were within normal ranges [1,2]. Constructional ability (Rey Complex Figure Copy) [13,14] was within the normal range. Although he could not remember his previous admission to the hospital, nor his stay in the intensive care unit, long-term memory, language, general semantic knowledge and autobiographical memory were unimpaired, and he had good insight. His behavior was appropriate and he was fully adapted to social life.

## 3. Discussion

Despite adequate medical treatment, some HSE patients show tentorial herniation of one or both temporal lobes during the course of illness. Both a decompressive craniotomy, with anterior temporal lobe resection, and a decompressive hemicraniectomy can be effective in controlling intractable, elevated intracranial pressure in HSE. In the literature, there have been few cases reports of the surgical management of HSE with herniation, dealing with the outcomes in term of long-term quality of life and function.

Taferner et al. [17] reported the long-term sequelae (1.5–8 years after craniectomy) of four cases with HSE and confirmed its appropriateness, as it led to full cognitive recovery, resocialization, and reintegration into professional life

Ebel et al. [6] suggested that not only a decompressive craniotomy but also a partial resection of the temporal lobe may be of benefit for patients with tentorial herniation, because both decompression and reduction of infectious material with cystic tissue necrosis can be achieved; therefore, we preferred to perform a decompressive craniotomy with an anterior temporal lobe resection.

It is known that HSE survivors are commonly affected by severe cognitive deficits, attention problems, with frontal impairment, loss of memory, and behavioral changes. Kapur et al. reported [9] that long-term neuropsychological impairments were characterized by dense amnesia in 60% of cases and noticeable anterograde memory impairment the others. Furthermore, visual object recognition deficits and severe frontal impairment symptoms, such as confabulatory and dysexecutive syndrome, as well as violent and sexually disinhibited behavior were reported [3,4].

We conclude that decompressive surgery for HSE with herniation signs can positively affect patient survival, with good outcomes in terms of cognitive function. Patients can benefit from anterior temporal lobe resection, not only in terms of neurological outcome, but also in cognitive profile. After the post-operative follow up period, the patients can survive without severe neurological or mental deficits. Moreover, the initial neurologic deficit seems to have no effect on the long-term clinical outcome.

## References

[1] A.L. Benton and M.W. Van Allen, Impairment of facial recognition in patient with cerebral disease, *Cortex* **4** (1968), 344–358.

[2] A.L. Benton, N.R. Varney and K.S. Hamsher, Visuospatial judgment: a clinical test, *Arch Neurol* **35** (1978), 364–367.

[3] F. Borgo, T.M. Sgaramella, B. Penello, R. L'Erario and V. Toso, A componential analysis of visual object recognition deficits in patients with herpes simplex virus encephalitis, *Brain Cogn* **43** (2000), 53–56.

[4] N. Del Grosso Destreri, E. Farina, E. Calabrese et al., Frontal impairment and confabulation after herpes simplex encephalitis: A case report, *Arch Phys Med Rehab* **83** (2002), 423–426.

[5] D. Delis, J. Kramer, E. Kaplan and B. Ober, *The California Verbal Learning Test*, The Psychological Corporation, San Antonio, Texas, 1987.

[6] H. Ebel, J. Kuchta, A. Balogh and N. Klug, Operative treatment of tentorial herniation in herpes encephalitis, *Childs' Nerv Syst* **15** (1999), 84–86.

[7] M. Folstein, S. Folstein and P. McHugh, "Mini-mental state." A practical method for grading the cognitive state of patients for the clinician, *J Psychiatr Res* **12** (1975), 89–198.

[8] D.A. Grant and E. Berg, *The Wisconsin Card Sort Test Random Layout: Directions for administration and scoring*, Wells Printing, Madison, Wisconsin, 1980.

[9] N. Kapur, S. Barker, E.H. Burrows et al., Herpes simplex encephalitis: long term magnetic resonance imaging and neuropsychological profile, *J Neurol Neurosurg Psychiatry* **57** (1994), 1334–1342.

[10] S. Karakas, *BILNOT battery: research and development of neuropsychological tests*, Ankara, Turkey: Dizayn Ofset, 2004.

[11] A. Luria, *Human Brain and Psychological Processes*, Harper&Row, New York, 1966.

[12] A. Mollahasanoglu, *Effects of age and education on a group of visual and verbal memory tests performance in normal subjects*. Istanbul University, Department of Psychology, Institute of Social Science (unpublished post-graduate thesis), 2002.
[13] E. Ozdeniz, *Effects of age and education variables on a group of right hemisphere and attention tests.* Istanbul University, Department of Psychology, Institute of Social Science. (unpublished post-graduate thesis), 2001.
[14] A. Rey, *L'examen clinique en psychologie*, Presses Universitaires de France, Paris, 1970.
[15] J. Stroop, Studies of interference in serial verbal reactions, *Exp Psychol* **18** (1935), 643.
[16] E. Taferner, B. Pfausler, A. Kofler et al., Craniectomy in severe life-threatening encephalitis: a report on outcome and long term prognosis of four cases, *Intensive Care Med* **27** (2001), 1426–1428.
[17] A. Tumac, *Effects of age and education on performance of some tests sensitive to frontal damage in normal subjects*. Istanbul University, Department of Psychology, Institute of Social Science (unpublished post-graduate thesis), 1997.
[18] D. Wechsler, *Wechsler Memory Scale-III. The psychological Corporation*, San Antonio, Texas, 1998.
[19] S. Weintraub, Neuropsychological Assessment of Mental State, in: *Principles of Behavioral and Cognitive Neurology*, 2nd edition, J. M-Marsel Mesulam, ed., New York, Oxford University Press, 2000, pp. 121–173.
[20] H.J. Yan, Herpes Simplex Encephalitis: The role of surgical decompression, *Surg Neurol* **57** (2002), 20–24.
